# Solid-Phase Synthesis and *In-Silico* Analysis of Iron-Binding Catecholato Chelators

**DOI:** 10.3390/ijms21207498

**Published:** 2020-10-12

**Authors:** Ranko Gacesa, Andrea A. P. Tripodi, Agostino Cilibrizzi, Antonella Leggio, Robert Hider, Vincenzo Abbate

**Affiliations:** 1 Institute of Pharmaceutical Science, King’s College London, London SE1 9NH, UK; r.gacesa@umcg.nl (R.G.); andrea.tripodi@agilent.com (A.A.P.T.); agostino.cilibrizzi@kcl.ac.uk (A.C.); robert.hider@kcl.ac.uk (R.H.); 2 Department of Gastroenterology and Hepatology, University of Groningen and University Medical Center Groningen, 9713 GZ Groningen, The Netherlands; 3 Department of Genetics, University of Groningen and University Medical Center Groningen, 9713 AV Groningen, The Netherlands; 4 Department of Pharmacy, Health and Nutritional Sciences, University of Calabria, 87036 Rende, Italy; antonella.leggio@unical.it; 5 Department of Analytical, Environmental and Forensic Sciences, King’s College London, London SE1 9NH, UK

**Keywords:** Siderophore, catechol, hexadentate, synthesis, iron(III) complexes, structure prediction

## Abstract

Siderophores are iron-complexing compounds synthesized by bacteria and fungi. They are low molecular weight compounds (500-1500 Daltons) possessing high affinity for iron(III). Since 1970 a large number of siderophores have been characterized, the majority using hydroxamate or catecholate as functional groups. The biosynthesis of siderophores is typically regulated by the iron levels of the environment where the organism is located. Because of their exclusive affinity and specificity for iron(III), natural siderophores and their synthetic derivatives have been exploited in the treatment of human iron-overload diseases, as both diagnostic and therapeutic agents. Here, solid-phase approach for the preparation of hexadentate, peptide-based tricatecholato containing peptides is described. The versatility of the synthetic method allows for the design of a common scaffolding structure whereby diverse ligands can be conjugated. With so many possibilities, a computational approach has been developed which will facilitate the identification of those peptides which are capable of providing a high affinity iron(III) binding site. This study reports an integrated computational/synthetic approach towards a rational development of peptide-based siderophores.

## 1. Introduction

Hexadentate iron(III) specific catecholato ligands have been extensively studied [1], for instance there is a large range of natural hexadentate chelators, termed siderophores, which are used by microorganisms to scavenge iron from the environment [2]. In addition synthetic hexadentate catecholato chelators have been designed to compete with natural siderophores, resulting in antimicrobial properties [3]; to scavenge toxic metals from the environment, for instance plutonium, [4] and to form high affinity complexes of Gd^4+^, Ga^3+^, In^3+^ for medical imaging [5,6,7]. The synthesis of such hexadentate ligands can be complicated in so far as often the synthetic routes consist of over ten consecutive reactions and the deprotection of oligodentate ligands is frequently time consuming and difficult to achieve complete deprotection [8,9]. Both these characteristics tend to lead to low yields. Furthermore, the determination of iron(III) affinity constants and pKa values for tricatecholato chelators are difficult by virtue of their extremely high values [10]. Thus the ability to predict iron(III) affinity trends of a series of theoretical catecholato ligands is likely to facilitate such work. Raymond and colleagues have previously reported studies on prediction models to rationalise the design of hexadentate catecholato iron ligands [11].

In order to simplify synthetic routes, we have developed a solid-phase approach for the preparation of peptide-based tricatecholate containing peptides. Fmoc amino acids were adopted, together with the coupling agents PyOxP/DIPEA, or OXYMA/DIC. Peptide deprotection was achieved with 20% *v/v* piperidine in DMF. BBr_3_ was utilized for the multiple demethylation of 2,3-dimethoxybenzoic acid (DBA), followed by semi-preparative HPLC purification. In addition, to rationally design improved peptide-based ligands, we also report here the use of Density Function Theory (DFT) [12] to predict the structure of iron complexes. We reasoned that the degree of deviation from the ideal iron coordination sphere could provide an indication of the stability of such complexes. From structural considerations, the angle of an ideal octahedral iron(III) d^2^sp^3^ hybrid should be 180° [13]. The sum of the 3 transverse angles in a perfect hexagon will be 180 × 3 = 540°. This can only be achieved by monodentate ligands such as OH^-^. When bidentate ligands coordinate iron(III), for instance catechol, the angle provided by each ligand is less than 180° due to the restricted bite distance of the two oxygens; for catechol the angle is 167° and thus the sum of the three transverse angles is 501.9° [14]. This deviation from 540° will lead to a decreased ∆H for complexation as the overlap of the orbitals cannot be maximized. When 3 such bidentate units are joined together by a molecular spacer, such octahedron distortion may become even larger leading to a further decrease in the enthalpy of complexation. We have compared the DFT-determined structures of a range of iron(III) siderophore complexes with their corresponding X-ray diffraction derived structures and found excellent agreement [15]. Consequently, we have used DFT techniques to investigate such differences in a range of known and hypothetical hexadentate chelators in the hope that we would be able to reliably predict the relative stabilities of such complexes in order to facilitate a targeted synthetic approach of new ligands in future work.

## 2. Results and Discussion

### 2.1. Synthesis of Tricatecholato Ligands Attached to a Peptide Backbone

**Figure 1:**
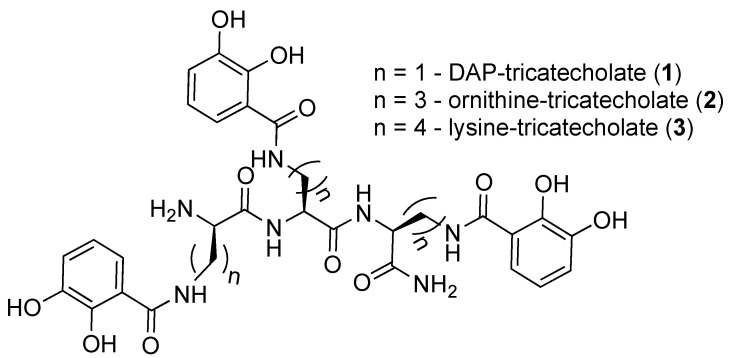
Structures of the synthesized tricatecholato-containing peptides.

**Figure 2:**
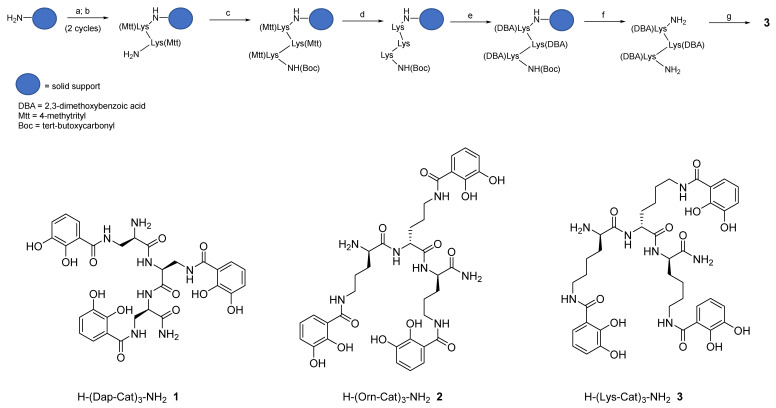
Schematic route for the synthesis of hexadentate peptide-chelator **3**: (a) Fmoc-Lys(Mtt)-OH, Oxyma, N,N’-diisopropylcarbodiimide (DIC), DMF; (b) 20% piperidine/DMF; (c) Boc-Lys(Mtt)-OH, Oxyma, DIC, DMF; (d) 1% TFA/DCM; (e) 2,3-dimethoxybenzoic acid (DBA), PyOxP/DIPEA, DMF; (f) TFA; (g) BBr_3_/DCM.

Hereby we describe a rapid, efficient, and versatile solid-phase approach for the preparation of a small library of peptide-based tricatecholate containing peptides (Figure 1). A generic synthetic route for the preparation of a lysine-based tricatechol (**3**) is presented in Figure 2. NovaPEG Rink Amide resin (hereby used to prepare carboxy amidated peptides) was coupled with the orthogonally protected Fmoc-Lys(Mtt)-OH using OXYMA/DIC chemistry [16], followed by removal of the Fmoc group with 20% piperidine/DMF. The process can be repeated as many times as required, for instance three acylations can be conducted for the preparation of a hexadentate scaffold. Boc-Lys(Mtt)-OH was preferred as the amino-terminal amino acid since the Boc group could be simultaneously removed together with the cleavage of the peptide from the solid support under high TFA concentration conditions. Boc-Lys(Mtt)-OH can be coupled at the N-terminus just before a multiple N-methyltrityl- (Mtt-) side chain deprotection is required. N-terminal Fmoc-protected amino acid was employed whenever further chemistry was conducted at the amino-terminus site, such in the case of N-terminal acetylation, N-terminal further derivatization, or for the synthesis of the octadentate ligands, which is the subject of future investigations in our laboratory.

The above procedure was repeated for the preparation of tricatecholate-based peptides where the catechol groups were linked to the peptide scaffold via a diaminopropionic acid (DAP) and ornithine group for compounds (**1** and **2**), respectively (Figure 2). Accordingly, a “mixed amino acid” approach could be undertaken if mixtures of side arm lengths are desired, such is the case of H-(Lys-Cat)_2_(Dap-Cat)-NH_2_ (**19**) (Table 2, Figure 4).

Following solid-phase assembly of the scaffolding structure, selective removal of the Mtt group was achieved using 1% TFA/DCM. Subsequently, coupling with 2,3 dimethoxybenzoic acid (DBA) was most efficiently achieved using PyOxP/DIPEA in DMF [17], as in our hands the multiple acylation of DBA residues were incomplete using standard peptide coupling conditions. Following removal of the protected-catecholate (DBA) peptide from the solid support, demethylation of the DBA residues was accomplished under BBr_3_/DCM conditions. The crude compound was finally purified using semi-preparative reverse-phase (RP) HPLC-DAD-MS and further characterized via analytical RP-HPLC-DAD, HRMS, and ^1^H NMR (see Supplementary Materials).

As mentioned above, the route is applicable for the assembly of “hybrid” chelators, where mixed arm catechols but also other chelator-types may be conjugated into the same scaffold. This can be achieved by selective deprotection of an orthogonal protecting group in the growing Fmoc-N-terminal peptide sequence followed by acylation with an appropriate carboxylic acid-containing chelator. Subsequently, the Fmoc-group is deprotected and the next Fmoc-amino acid coupled. The conjugation of another chelating unit can then be achieved by repeating one more time the above cycle. The preparation of a library of “hybrid” chelators is currently under investigation in our laboratory.

### 2.2. Analysis of iron(III) Complexes of Tricatecholato Structures

To predict improved synthetic siderophores that could be synthesized by our newly developed solid-phase method, we have investigated in-silico 5 siderophores, namely **4**–**8**, together with 4 siderophore analogues **9**–**12**. The two parameters, transverse angle sum and the roots mean square differences (RMSD) between the octahedral of oxygen atoms in the complex and a perfect octahedron (see Materials and Methods), were used to analyse the entire group of the 9 iron(III) complexes (Table 1).

**Figure 3:**
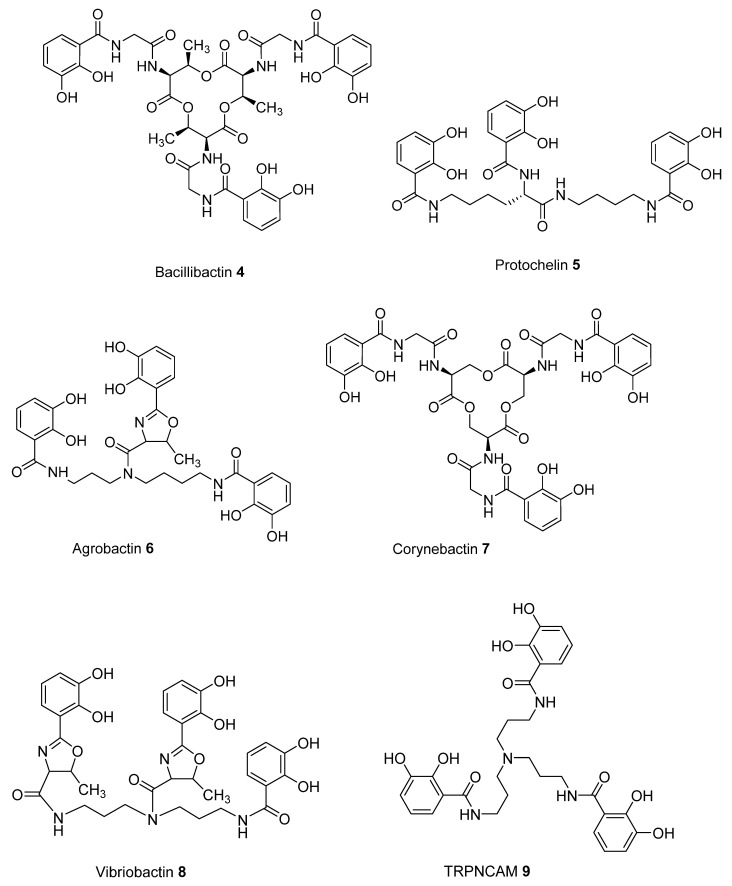

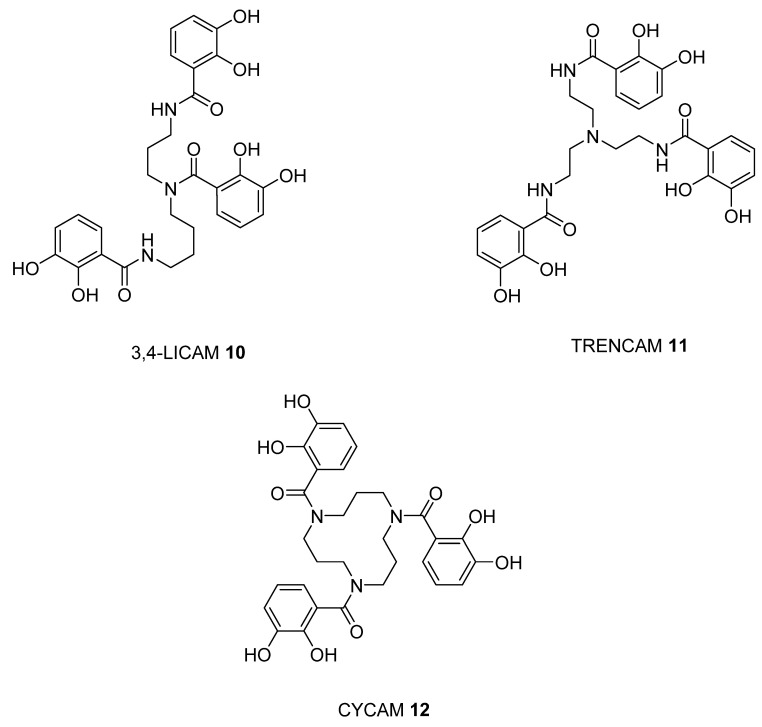

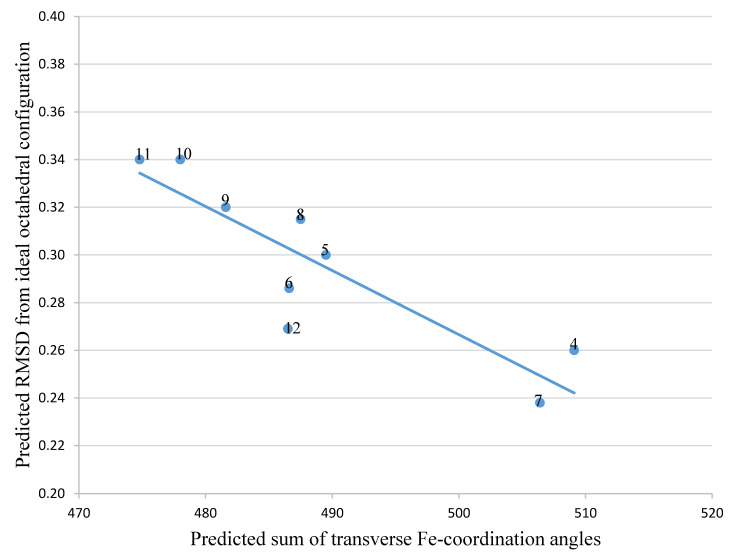
The relationship between predicted RMSD values and sum of transverse Fe-coordination angles for a series of siderophores and siderophore analogues.

Comparison of the 9 compounds demonstrated that only two, bacillibactin (**4**) and corynebactin (**7**), were located in the vicinity of the “500/0.250” cutoff values (see Materials and Methods) (Figure 3). Both compounds possess relatively long linking arms between the central triester ring and the catechol function; namely 5 atoms. The remaining siderophores **5**, **6** and **8** are clustered together and located well below the “500/0.250” cutoff values. Protochelin (**5**) has one short linking segment (2 atoms) and agrobactin (**6**) and vibriobactin (**8**) both possess rigid linking segments containing a heterocyclic ring. These factors clearly mitigate against the three coordinating catechol groups, forming an optimal stereochemistry around iron(III).

With the catechol siderophore analogues (**9**, **10**, **11**, and **12**), none are located close to the cutoff values, all the structures apparently providing a non-ideal geometry for optimal iron(III) coordination. Thus tripodal nitrogen based backbones form less suitable octahedral stereochemistry for the iron(III) binding site in comparison to amide-based siderophores, for instance **4** and **7**.

### 2.3. Analysis of iron(III) Complexes of Hypothetical Tricatecholato Structures

**Figure 4:**
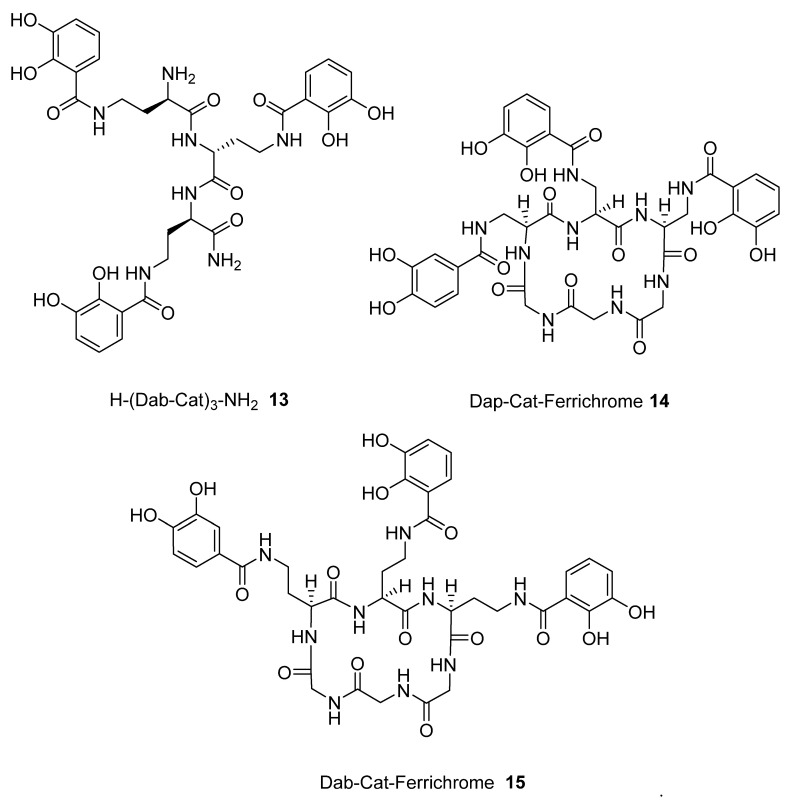

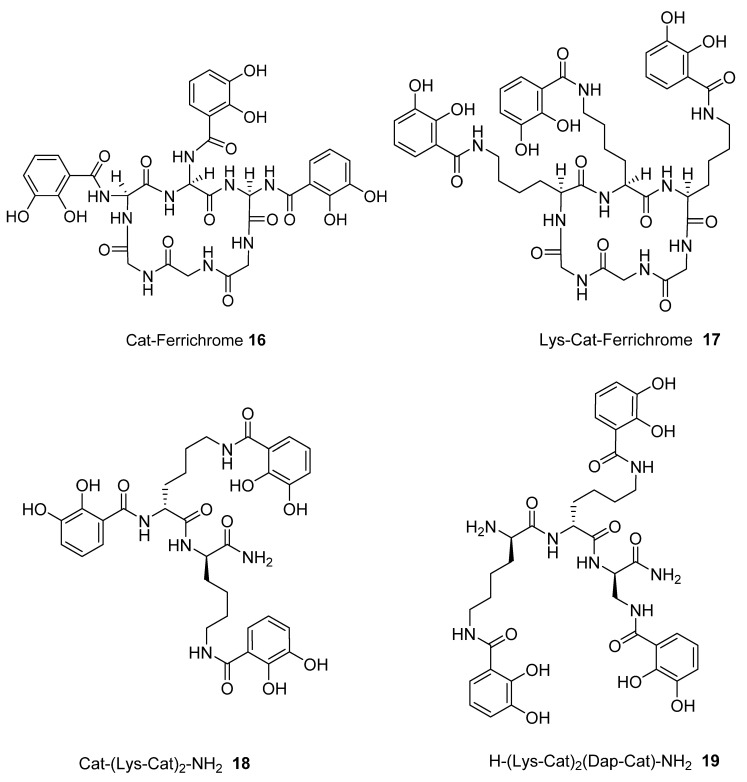

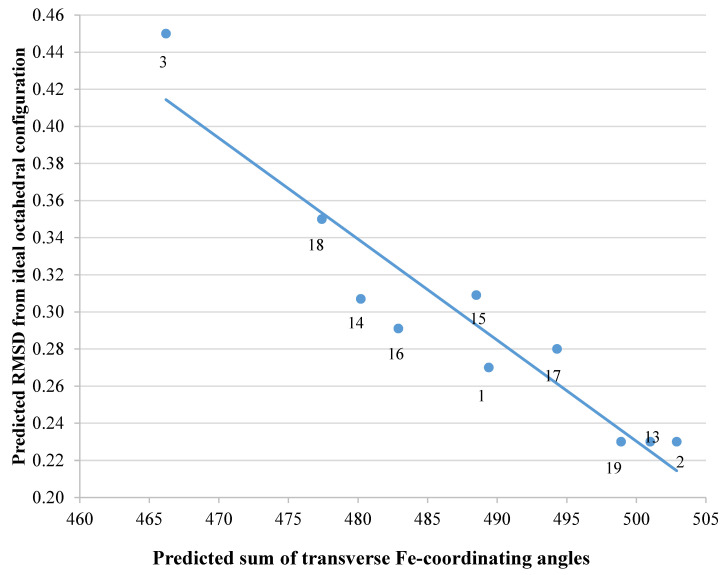
The relationship between predicted RMSD values and sum of transverse Fe-coordination angles for a series of peptide based tricatecholate ligands.

We subsequently analysed the structures of ten hypothetical siderophore analogues which are based on various peptide linking groups (Table 2). Again, all these structures form a linear relationship between the two parameters, angle sum and RMSD (Figure 4). Two of the ligands (**2** and **13**) exceed the “500/0.250 cutoff” values but the rest do not. Based on this analysis, when three adjacent catechol-bearing arms are attached to a cyclic hexapeptide in the style of ferrichrome, namely **15**–**18**, irrespective of the length of the linking group, a highly favorable iron(III) coordination site cannot be created and the two parameters, angle sum and RMSD, fall below the “500/0.250 cutoff” values (Figure 4). In contrast, when three catechol moieties are attached to a short linear peptide, **2** and **13**, then a superior iron(III) binding site can be achieved (Table 2 and Figure 4). The stereochemistry of these molecules is critically dependent of the length of the amino acid chain, thus 1 and 4 methylene groups fail to yield an ideal iron(III) coordination site, whereas 2 and 3 generate such a site. Mixtures of side arm lengths are also capable of leading to compounds with a superior octahedral iron(III) binding site, thus although H-(Dap-Cat)_3_-NH_2_ (**1**) and H-(Lys-Cat)_3_-NH_2_ (**3**) fail to generate a high-affinity site for iron(III), H-(Lys-Cat)_2_(Dap-Cat)-NH_2_ (**19**) is capable of generating such a site (Table 2, Figure 4).

As indicated in the Introduction, the measurement of iron(III) affinity constants for hexadentate tricatechols is extremely difficult and we have been unable to determine accurate values for ligands (**2** and **13**) at the present time. However, we have used the method introduced by Ma et al. for the determination of pFe^3+^ values for multidentate ligands by fluorescence [18] and this analysis has preliminarily yielded a high pFe^3+^ value for the prototype ligand **2** (>25). Further work is currently being conducted both for the preparation of siderophore analogue **13**, and for the accurate determination of these ligands’ pFe^3+^ values to confirm our in-silico predictions.

## 3. Materials and Methods

### 3.1. Structural Analysis of iron(III) Complexes

Structures of iron chelating compounds were generated using Avogadro molecule editor and visualizer [19] and manually inspected to confirm appropriate positions of chiral groups based on known 3d structures of similar iron-chelators. Approximate geometry was obtained via Universal Force Field (UFF) [20] based energy minimization in Avogadro. Further optimization of structure was performed using Density Functional Theory (DFT) [21] via the ORCA quantum chemistry package (version 3.0.3, Max Planck Institut für Kohlenforschung., Mulheim an der Ruhr, Germany) [22]. Simulations were performed using the exchange functional of Tao et al. (TPSS) [23], previously benchmarked as a highly performing functional for iron complex geometries [20]. ZORA-DEF2-SVP basis set was used on Carbon and Hydrogen atoms and polarized triple-zeta basis set with relativistic corrections (def2-TZVP basis) was used on other atoms. Resolution of identity (RI) approximation was used to increase calculation speed, with UKS correction for handling open-shell broken-symmetry singlet state and Grimmer’s DFT-3D dispersion correction (D3BJ). Self-Consistent Field (SCF) calculations were performed using slow convergence, large grid (GRID 5, FINAL GRID 6), and parameters for tight geometry, optimization (TightOpt, Very tight SCF convergence).

Effects of RI approximation and basis sets were assessed by comparative simulation on five randomly chosen compounds, using def2-TZVP basis on all atoms and no approximations and differences in resulting iron coordinating angles were less than 0.1 degree. Visualization of results and calculations of iron-coordinating angles and relative mean square distance (RMSD) deviations from optimal octahedral geometry were performed using USCF Chimera toolkit, version 1.10.02 (University of San Francisco, San Francisco, CA, USA) [24].

An analysis of eight X-ray structures of siderophores were used to generate both the sums of the 3 octahedral O-Fe-O angles and the roots mean square differences (RMSD) from a perfect octahedron. There was a strong linear correlation between these two parameters. The high iron(III) affinity ligands are present in the graph regains where the total sum of the transverse angles is greater than 500 and the RMSD value is less than 0.25 [15]. Thus these two values were selected as cutoff values used to distinguish between potentially useful hexadentate structures and structures likely to generate a suboptimal coordination site for iron(III).

### 3.2. Materials

Nova PEG Rink Amide resin, Fmoc-Lys(Mtt)-OH, Fmoc-Orn(Mtt)-OH and Fmoc-Dap(Mtt)-OH were purchased from Novabiochem (Hohenbrunn, Germany). DMF anhydrous, 99.8 %, was purchased from Sigma Aldrich (Steinheim, Germany) and was kept constantly under a steady stream of high purity nitrogen. 2,3-dimethoxybenzoic acid (DBA), N, N’-diisopropylcarbodiimide (DIC) and ethyl hydroxyimino cyanoacetate (OXYMA), piperidine, trifluoroacetic acid (TFA), triisopropylsilane (TIPS) and phenol were purchased from Sigma Aldrich (Steinheim, Germany). N, N’-diisopropylethylamine (DIPEA) was purchased from TCI Europe and Fluka (Buchs, Switzerland). Methanol, acetonitrile, dichloromethane (DCM) and diethylether were purchased from Fisher Scientific UK (Leichestershire, UK). Boron Tribromide (BBr_3_) (1M in DCM) was purchased Alfa Aesar, (Heysham, UK). PyOxP was synthesized according to a reported method [15].

### 3.3. Solid Phase Synthesis

NovaPEG Rink Amide resin (0.5g, 0.00049 mol/g) was placed into an empty fritted polypropylene column, which allows washing of the resin and removal of reagents used during the synthesis. The resin was firstly washed with DMF and then swollen with dry DMF for about 25 min. Subsequently, addition of the Fmoc amino acid to the resin was performed, together with PyOxP and DIPEA in a molar ratio of 1:3:3:6 (resin:Fmoc-amino acid: PyOxP: DIPEA), and the mixture was incubated in dry DMF overnight with gentle shaking. The subsequent couplings were conducted using OXYMA and DIC (1:3:2:2 ratio resin:Fmoc-amino acid:OXYMA:DIC). After overnight incubation, the resin was washed with dry DMF and MeOH and coupling monitored by colorimetric picrylsulphonic acid test [25].

For DBA coupling, the Mtt-free peptide-resin was treated with DBA, PyOxP, and DIPEA in DMF with a resin/reagent molar ratio of 1:3:3:6, leaving the reaction to gently stir overnight. Subsequently, the resin was washed under vacuum with DMF and MeOH and checked using picrylsulphonic acid test.

### 3.4. Deprotection Reactions

The Fmoc-deprotection step was achieved using a solution of 20% *v/v* piperidine in DMF. The resin was gently shaken with the piperidine solution for 10 min and then washed multiple times with DMF and then with MeOH under vacuum. Picrylsulphonic acid test was used again to qualitatively confirm deprotection.

The acid-labile 4-methyltrityl (Mtt) group was selectively removed using excess solution of 1% (*v/v*) TFA in DCM, with the addition of triisopropylsilane (TIPS) (2% *v/v*) to quench the trityl cations, at room temperature, incubating at least five times for 5 min. The methyltrityl cations produce an intense yellow color in DCM/TFA solution [26] due to the trityl carbonium ion chromophore when the protecting group is removed under mild acidic conditions. This procedure needed to be repeated until the solution became colorless. Each TFA/DCM treatment started by previously washing the resin with DMF, MeOH and finally DCM under vacuum.

For the multiple demethylation of DBA, the freeze-dried cleaved product was suspended in dry DCM (~ 5 mL/ 20–30 mg material) and transferred to the reaction flask previously washed with conc. HCl. The flask was allowed to be cooled using an ice/water bath and flushed with nitrogen. After few minutes BBr_3_ 1 M in DCM (~3 mL for 20–30 mg material) was added dropwise over ice and nitrogen and the mixture was stirred overnight at room temperature, monitoring the reaction by RP-HPLC and low-res MS (a drop of solution was quenched with 100 uL MeOH for 1 h prior to HPLC and MS analysis). Once the deprotection was deemed complete, 10 mL of MeOH were added dropwise to the solution chilled in an ice/water bath and stirred for 1 h at room temperature. The solution was transferred to a round-bottom flask and evaporated to dryness. The resulting product was treated with MeOH and precipitated using excess ice-cold diethyl ether, centrifuged, isolated, and dissolved in HPLC in mobile phase. The crude product was finally lyophilized for 24 h prior to being subjected to semi-preparative HPLC purification.

### 3.5. Peptide-Resin Cleavage Procedure

The cleavage solution was prepared with the following reagents: TFA/phenol/H_2_O/TIPS (87.5/5/5/2.5). Three ml of cleavage cocktail were added to 100 mg of the peptide-resin and the reaction was incubated for 3 h at room temperature. The solution was filtered into a falcon tube and the resin was washed with a small volume of cleavage cocktail. Crude peptides were subsequently precipitated using 10x excess ice-cold diethyl ether. The mixture was centrifuged at 4000 rpm for 5 min and the precipitate was washed in an excess of fresh ice-cold diethyl ether and centrifuged again. Before the pellet was dissolved with HPLC mobile phase (50% ACN/50% water/0.1 TFA) for the analysis, evaporation of residues of diethyl ether was performed using a gentle stream of N_2_ to remove any trace of organic solvent. The pellet was dissolved in 10 mL of a solution 50% ACN/50% H_2_O containing 0.1 % TFA, part of the solution was taken for the HPLC and MS analysis, whereas all the remaining solution containing the crude product was lyophilized for 24 h.

Tricatecholate-based peptide **1**. ^1^H NMR (400 MHz, CD_3_OD+D_2_O) *δ* 7.16–7.28 (m, 3H, Ar), 6.90–6.94 (m, 3H, Ar), 6.66–6.74 (m, 3H, Ar), 4.65 (t, 1H, COC*H*NH), 4.14 (t, 1H, COC*H*NH), 3.87 (d, 6H, 3 x NCH*CH_2_*), 3.73–3.78 (m, 1H, COC*H*NH). Calculated m/z for [M+H]^+^ = 684.2260; found = 684.2272. For detailed spectra see Supplementary Materials Figures S1 and S4.

Tricatecholate-based peptide **2**. ^1^H NMR (400 MHz, D_2_O) *δ* 7.08 (dd, 1H, Ar), 6.99 (dd, 1H, Ar), 6.95 (dd, 1H, Ar), 6.91 (td, 1H, Ar), 6.83 (dd, 1H, Ar), 6.63–6.73 (m, 4H, Ar), 4.37 (t, 1H, COC*H*NH), 4.30 (t, 1H, COC*H*NH), 4.06 (t, 1H, COC*H*NH), 3.12–3.34 (m, 6H, 3 x NCH_2_), 1.45–1.91 (m, 12H, 3 x CH*(CH_2_)_2_*). Calculated m/z for [M+H]^+^ = 768.3199; found = 768.3211. For detailed spectra see Supplementary Materials Figures S2 and S5.

Tricatecholate-based peptide **3**. ^1^H NMR (400 MHz, CD_3_OD+D_2_O) *δ* 7.36 (d, 1H, Ar), 7.18–7.26 (m, 2H, Ar), 7.04–7.09 (m, 1H, Ar), 6.88–6.93 (m, 2H, Ar), 6.81–6.85 (m, 1H, Ar), 6.65–6.73 (m, 2H, Ar), 4.27–4.38 (m, 2H, 2 x COC*H*NH), 3.69 (d, 3H, COC*H*NH + NCH_2_), 3.35–3.40 (m, 4H, 2 x NCH_2_), 1.33–1.98 (m, 18H, 3 x CH*(CH_2_)_3_*). Calculated m/z for [M+H]^+^ = 810.3668; found = 810.3691. For detailed spectra see Supplementary Materials Figures S3 and S6.

### 3.6. Method for Determination of pFe^3+^

A recently reported fluorescent method was used to determine the pFe^3+^ values of the prototype ligand **2**. [18]. The pFe^3+^ was determined based on acid dissociation constants of the free ligand (pKa) and stability constants of the iron–ligands complex (log β3). Although the hexadentate probes are highly selective for iron(III), they are also responsive to iron(II) which is rapidly oxidized after reacting with the probe to form the iron(III) complexes.

### 3.7. Instrumentation

Lyophilization was performed using liquid nitrogen and a Thermosavant freeze dryer (Edwards, Crawley, UK).

Analytical RP-HPLC was conducted on a HP1050 HPLC system (Agilent Technologies, Waldbronn, Germany) equipped with an autosampler, a quaternary pump and a Diode-Array Detector (DAD). The flow rate was 0.2 mL/min and the eluents were monitored at wavelengths between 214–281 nm. A linear gradient of mobile phase B (acetonitrile containing 0.1% TFA) over mobile phase A (0.1% TFA in water) from 0–90% B in 20 min was performed. Data were collected and analyzed using ChemStation software (Agilent Technologies, Waldbronn, Germany). For the HPLC analysis, a C18-SB Zorbax 3.5 micron (2.1 × 100 mm) was used.

The purification of the peptides was performed using a Waters Autopurification semi-preparative reverse-phase high performance liquid chromatography (HPLC) system (Waters, Milford, MA, USA), coupled with a dual DAD and single quadrupole MS detector. The column was a XTerra Prep MS C18- 5µm, 10 × 100 mm. Eluents were collected automatically using mass-triggered collection. The mobile phases were water/0.1% TFA (A) and 90% ACN and 10% mobile phase A (B) and a linear gradient of B over A over 30 min at a flow rate = 7 mL/min was used to perform the semipreparative HPLC purification. Each collected fraction was analyzed via analytical RP-HPLC-DAD and low-resolution MS via direct infusion. Pure/identical fractions were pooled and lyophilized for 24 h. Low-resolution MS were obtained using a ZQ Micromass 2000 (Waters, Milford, MA, USA), operating with either positive or negative ESI mode. ^1^H NMR spectra were recorded using an internal deuterium lock at ambient probe temperatures on a Bruker Ascend^TM^-400 (400 MHz) (Bruker, Rheinstetten, Germany) instrument. Data were referenced to the residual non-deuterated solvent peak (i.e., MeOH or H_2_O) and analyzed for chemical shift, multiplicity, integration, assignment, and coupling constants. Assignments were determined either based on unambiguous chemical shift or coupling pattern, or by analogy to fully interpreted spectra for analogous compounds. High-Resolution Mass Spectra (HRMS) were conducted on an Exactive Orbitrap Mass Spectrometer (Thermo Fisher Scientific, Bremen, Germany) operating in positive electrospray ionization mode.

## 4. Conclusions

A facile and versatile solid-phase synthetic approach is described for the preparation of hexadentate, tricatecholato-based peptide siderophores. In addition, a computational approach was developed to facilitate the identification of peptides which are capable of providing a high affinity iron (III) binding site. This work enables the design of scaffolding structures on which diverse ligands can be conjugated. These two developments will greatly facilitate the development of future synthetic siderophore analogues.

## Figures and Tables

**Table 1:** Siderophore analogues.

Ligand in FeL Complex	Sum of Angles ^2^	RMSD ^1^
**4**	509.1	0.260
**5**	489.5	0.300
**6**	486.6	0.286
**7**	506.4	0.238
**8**	487.5	0.315
**9**	481.6	0.320
**10**	478.0	0.340
**11**	474.8	0.340
**12**	486.5	0.269

^1.^ Roots mean square differences (RMSD)—refers to the comparison of octahedra resulting from the six ligating oxygen atoms. ^2.^ Sum of angles = sum of the tree transverse angles.

**Table 2:** Hypothetical catechol siderophore analogues.

Ligand in FeL Complex	Sum of Angles ^2^	RMSD ^1^
**1**	489.4	0.270
**2**	502.9	0.230
**3**	466.2	0.450
**13**	501.0	0.230
**14**	480.2	0.307
**15**	488.5	0.309
**16**	482.9	0.291
**17**	494.3	0.280
**18**	477.4	0.350
**19**	498.9	0.230

^1.^ RMSD—refers to the comparison of octahedra resulting from the six ligating oxygen atoms. ^2.^ Sum of angles = sum of the tree transverse angles.

## Supplementary Materials

The following are available online at https://www.mdpi.com/1422-0067/21/20/7498/s1, Figure S1: Structures and elemental compositions of tricatecholate-based peptide **1** and its protonated form (A); HPLC-DAD/254nm (B), low res ESI+ MS (C) and ESI+ HRMS (D) (calculated m/z for [M+H]^+^ = 684.2260; found = 684.2272), Figure S2: Structures and elemental compositions of tricatecholate-based peptide **2** and its protonated form (A); HPLC-DAD/254nm (B), low res ESI+ MS (C) and ESI+ HRMS (D) (calculated m/z for [M+H]^+^ = 768.3199; found = 768.3211), Figure S3: Structures and elemental compositions of tricatecholate-based peptide **3** and its protonated form (A); HPLC-DAD/254nm (B), low res ESI+ MS (C) and ESI+ HRMS (D) (calculated m/z for [M+H] ^+^ = 810.3668; found = 810.3691), Figure S4: ^1^H NMR of tricatecholate-based peptide **1** (in CD_3_OD+D_2_O), Figure S5: ^1^H NMR of tricatecholate-based peptide **2** (in D_2_O), Figure S6: ^1^H NMR of tricatecholate-based peptide **3** (in CD_3_OD+D_2_O).

## Abbreviations

DFT	Density Function Theory
RMSD	roots mean square difference
OXYMA	ethyl hydroxyimino cyanoacetate
DIC	N,N’-diisopropylcarbodiimide
DBA	2,3-dimethoxybenzoic acid
PyOxP	*O*-[(1-cyano-2-ethoxy-2-oxoethylidene)amino]-oxytri(pyrrolidin-1-yl)phosphonium hexafluorophosphate
DIPEA	N,N’-diisopropylethylamine
UFF	Universal Force Field
