# Supplementary material for: Solid-Phase Synthesis and In-Silico Analysis of Iron-Binding Catecholato Chelators
Source: Int J Mol Sci. 2020 Oct 12;21(20):7498. doi: 10.3390/ijms21207498 (PMC7593911; doi:10.3390/ijms21207498)
Supplement: Supplementary file 1 [file ijms-21-07498-s001.docx]

**Supplementary material for**

**Solid-phase synthesis and *in silico*analysis of iron-binding catecholato chelators**

Ranko Gacesa ^1,2^, Andrea A.P. Tripodi ^1^, Agostino Cilibrizzi ^1^, Antonella Leggio ^3^, Robert Hider ^1^, Vincenzo Abbate ^4^*

Affiliations

^1^ Institute Pharmaceutical Science, King’s College London

^2^ University of Groningen and University Medical Center Groningen, Department of Gastroenterology and Department of Genetics, Groningen, the Netherlands

^3^ Department of Pharmacy, Health and Nutritional Sciences, University of Calabria, Rende, Italy

^4^ Department of Analytical, Environmental and Forensic Sciences, King’s College London, London, UK

^*^  [vincenzo.abbate@kcl.ac.uk](mailto:vincenzo.abbate@kcl.ac.uk); Tel: +44-20-7848-84489

S1 A

S1 D

**Figure S1:** Structures and elemental compositions of tricatecholate-based peptide **1** and its protonated form (A); HPLC-DAD/254nm (B), low res ESI+ MS (C) and ESI+ HRMS (D) (calculated m/z for [M+H]^+^ = 684.2260; found = 684.2272).

S2 A


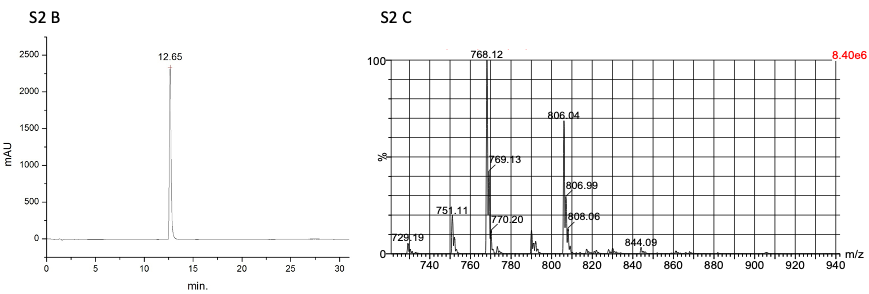

S2 D

**Figure S2:** Structures and elemental compositions of tricatecholate-based peptide **2** and its protonated form (A); HPLC-DAD/254nm (B), low res ESI+ MS (C) and ESI+ HRMS (D) (calculated m/z for [M+H]^+^ = 768.3199; found = 768.3211).

S3 A

**Figure S3:** Structures and elemental compositions of tricatecholate-based peptide **3** and its protonated form (A); HPLC-DAD/254nm (B), low res ESI+ MS (C) and ESI+ HRMS (D) (calculated m/z for [M+H] ^+^ = 810.3668; found = 810.3691).


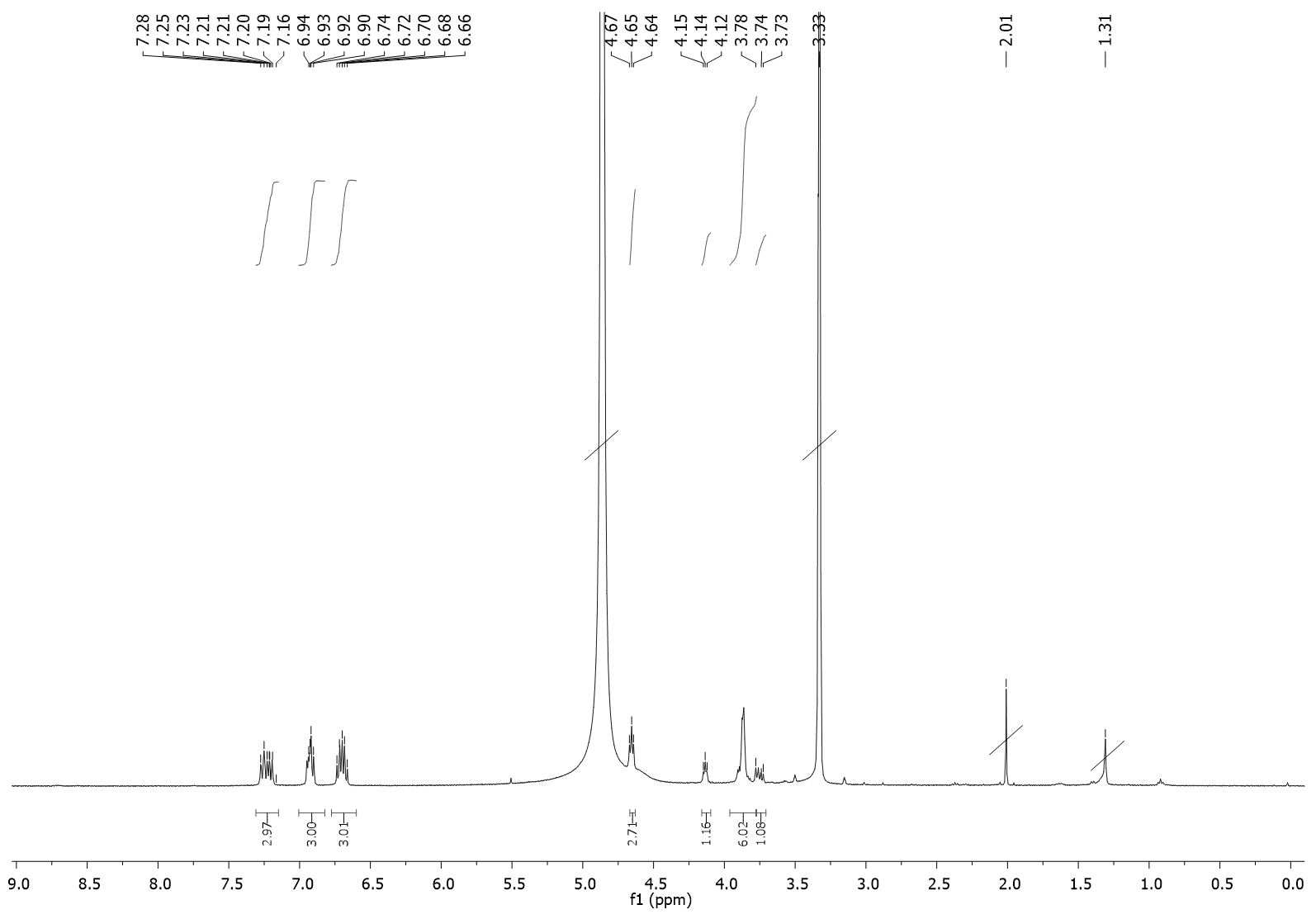


**Figure S4:** ^1^H NMR of tricatecholate-based peptide **1** (in CD_3_OD+D_2_O).

**
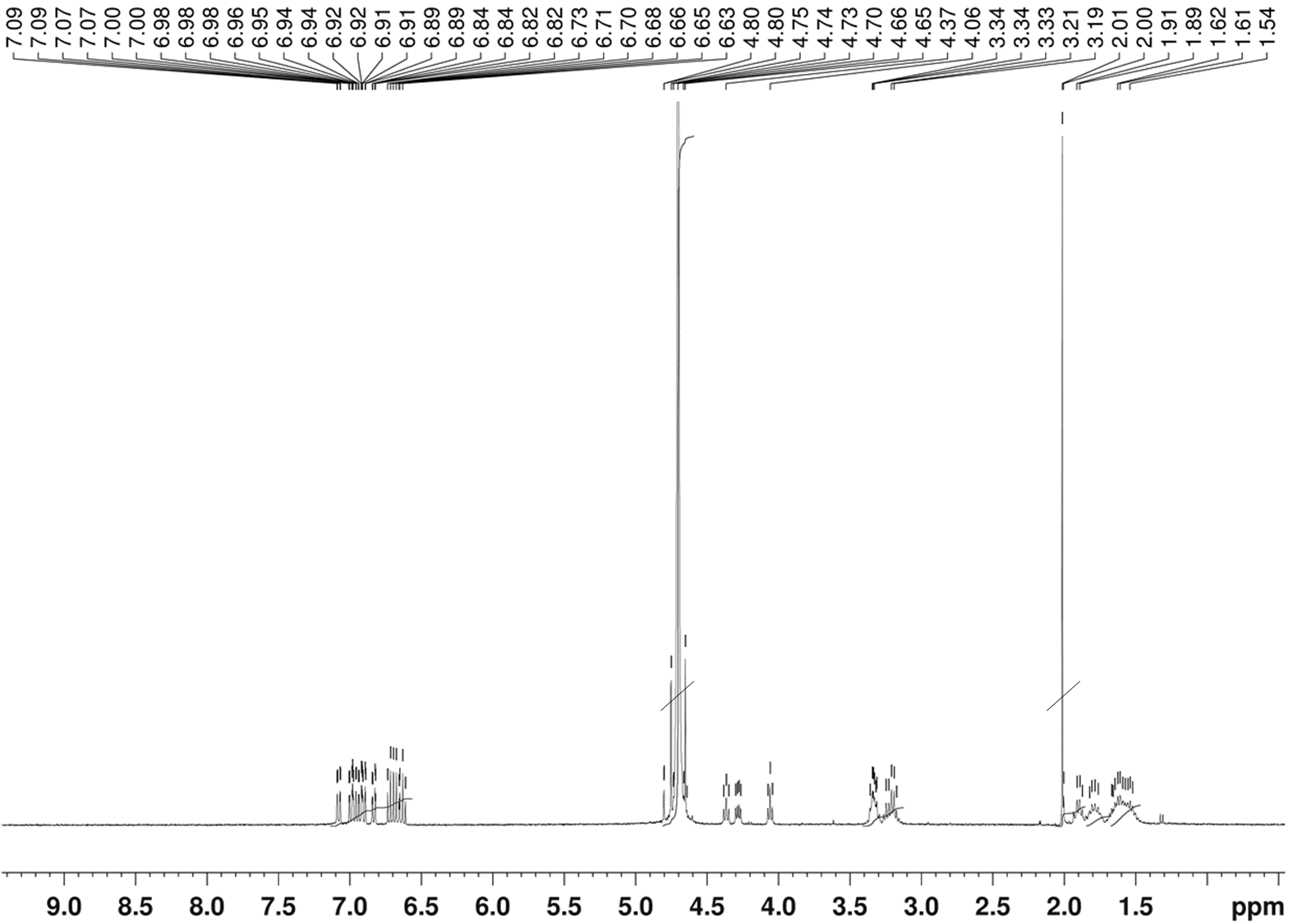
**

**Figure S5:** ^1^H NMR of tricatecholate-based peptide **2** (in D_2_O).


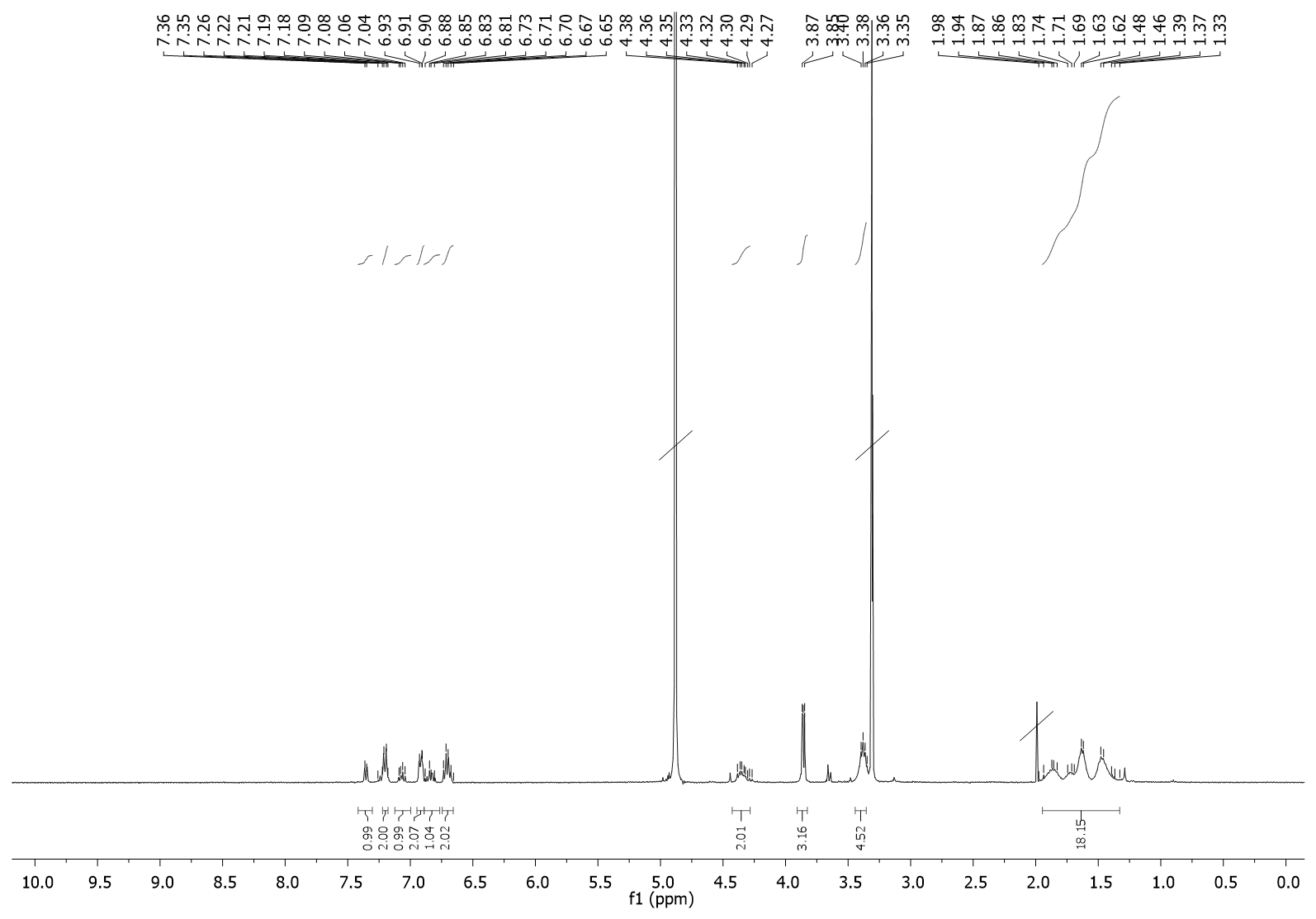


**Figure S6:** ^1^H NMR of tricatecholate-based peptide **3** (in CD_3_OD+D_2_O).
